# A novel *ITGA2B* double cytosine frameshift variant (c.1986_1987insCC) leads to Glanzmann's thrombasthenia in a cat

**DOI:** 10.1111/jvim.17030

**Published:** 2024-03-01

**Authors:** Victor N. Rivas, Avalene W. K. Tan, Meg Shaverdian, Nghi P. Nguyen, Jalena R. Wouters, Joshua A. Stern, Ronald H. L. Li

**Affiliations:** ^1^ Department of Medicine and Epidemiology, School of Veterinary Medicine University of California‐Davis Davis California USA; ^2^ Department of Clinical Sciences, College of Veterinary Medicine North Carolina State University Raleigh North Carolina USA; ^3^ Department of Surgical and Radiological Sciences, School of Veterinary Medicine University of California‐Davis Davis California USA

**Keywords:** congenital, feline, glycoprotein IIb/IIIa, macrothrombocytopenia, precision medicine, thrombopathia

## Abstract

**Background:**

Glanzmann's thrombasthenia (GT) is a congenital platelet disorder affecting approximately 1:1 000 000 people globally and characterized by impaired platelet aggregation and clot retraction. Autosomal recessive, loss‐of‐function, variants in *ITGA2B* or *ITGB3* of the αIIbβ3 receptor cause the disease in humans. A cat affected by Glanzmann's and macrothrombocytopenia was presented to the UC Davis VMTH.

**Hypothesis/Objectives:**

Severe thrombopathia in this cat has an underlying genetic etiology.

**Animals:**

A single affected patient, 2 age‐matched clinically healthy controls, and a geriatric population (n = 20) of normal cats.

**Methods:**

Physical examination and clinical pathology tests were performed on the patient. Flow cytometry and platelet aggregometry analyses for patient phenotyping were performed. Patient and validation cohort gDNA samples were extracted for Sanger sequencing of a previously identified *ITGA2B* (c.1986delC) variant. Reverse transcriptase PCR was performed on patient and healthy control PRP samples to verify *ITGA2B* variant consequence.

**Results:**

A novel c.1986_1987insCC autosomal recessive variant in *ITGA2B* was identified. This variant was absent in a population of 194 unrelated cats spanning 44 different breeds. Complete loss of ITGA2B transcript and protein expression was verified by RT‐PCR and flow cytometry, explaining the underlying etiology of GT, and likely macrothrombocytopenia, in this cat.

**Conclusions and Clinical Importance:**

This study emphasizes the role of precision medicine in cardiovascular disease of cats and identified yet another variant that may be of utility for screening in the feline population. This study provides a small‐volume, standardized, successful protocol for adequate platelet RNA isolation and subsequent molecular assessment of gene expression in cats.

**Abbreviations**
*ACTB*
: *beta (β)‐actin*
ADP: adenosine diphosphate
aPTT: activated partial thromboplastin time
AUC: area under the curve
CBC: complete blood count
DSH: domestic short hair
EVA: European Variation Archive
GT: Glanzmann's thrombasthenia
*ITGA2B*
: *integrin subunit alpha‐IIb*
*ITGB3*
: *integrin subunit beta‐3*
LOF: loss‐of‐function
MFI: median fluorescent intensity
MOI: mode of inheritance
NMD: nonsense‐mediated decay
PRP: platelet‐rich plasma
PT: prothrombin time
RT‐PCR: reverse transcriptase‐polymerase chain reaction

## 1 INTRODUCTION

Primary inherited platelet disorders (thrombocytopathies) are rare, affecting an estimated 1:10 000‐1:1 000 000 humans.^1^ At least 75 variants explaining approximately 60 different types of thrombocytopathies have been described in humans with associated molecular defects in hemostatic processes.^2^ In the general human population, the prevalence of such variants remains low; approximately 0.3% of individuals harbor at least 1 copy of a pathogenic variant leading to severe bleeding tendencies and commonly accompanied by severe pleiotropic effects.^1^, ^3^

Glanzmann's thrombasthenia (GT) is the most widely understood and recognized congenital platelet disorder in small animals. This inherited platelet disorder affects roughly 1 in every 1 million people globally and is characterized by impaired platelet aggregation and clot retraction.^4^ Autosomal recessive, loss‐of‐function (LOF), variants in either of the *integrin subunit alpha‐IIb* (*ITGA2B*) or *beta‐3* (*ITGB3*) of the platelet integrin αIIbβ3 receptor explains the disease in humans.^5^ In dogs, the disease is caused by variants of the same genes and is over‐presented in Great Pyrenees and Otterhounds.^6^, ^7^, ^8^ We recently identified a cytosine deletion variant (c.1986delC) in exon 18 of the *ITGA2B* gene using whole genome sequencing in a cat with GT. This cat experienced intermittent and spontaneous bleeding diatheses including epistaxis, hematuria after cystocentesis, and gastrointestinal bleeding. The mode of inheritance (MOI) of this first reported feline variant is autosomal recessive and results in a sequence frameshift with an implicated premature stop codon.^9^

Here, we report a case of a 21‐month‐old cat with a congenital platelet disorder. Given the patient history and suggestive underlying congenital disease, we hypothesized the severe thrombopathia was because of an underlying genetic etiology and set out to identify possible associated genetic variants. Our study emphasizes the role of precision medicine in cardiovascular disease of cats and identified yet another genetic variant that may be of utility for screening in the feline population.

## 2 MATERIALS AND METHODS

### 2.1 Ethics statement

All procedures were approved by the University of California‐Davis (Davis, California, USA) Institutional Animal Care and Use Committee (protocol #20095 and #21857) and carried out in accordance with guidelines and regulations; this study also was carried out in compliance with the ARRIVE guidelines.^10^ Written owner consent was obtained for all sample collections.

### 2.2 Case description

A 21‐month‐old, female spayed, domestic short hair (DSH) cat was referred to the Emergency Service of the Veterinary Medical Teaching Hospital of University of California‐Davis for further evaluation of recurrent episodes of respiratory distress. Before referral, the cat had a 15‐month history of waxing and waning respiratory signs characterized by intermittent open mouth breathing, coughing, increased respiratory effort, and exercise intolerance. Intermittent treatments with amoxicillin‐clavulanic acid, prednisolone, fluticasone, and doxycycline were prescribed. Four days before referral, the cat was presented to an emergency clinic for hemoptysis and tachypnea. At that time, coarse pulmonary crackles and referred upper airway noises were noted. Thrombocytopenia (platelet count of 30 000/μL) and anemia (hematocrit of 28%) were noted on CBC. Thoracic radiographs indicated interstitial pulmonary infiltrates in the right middle lung lobe, tracheal narrowing within the cranial mediastinum, and diffuse soft tissue opacity in the cranial mediastinum suggestive of fluid accumulation within the trachea and mediastinum. Clotting times consisting of prothrombin time (PT) and activated partial thromboplastin time (aPTT) were within normal ranges. The cat was monitored in oxygen and discharged with oral amoxicillin‐clavulanic acid. Two days later, the cat was presented to the family veterinarian for ongoing anorexia, presence of petechia, and ecchymoses.

### 2.3 Day one physical examination & clinical pathology results

On presentation, the cat had noticeable stridor upon gentle restraint. Ecchymoses were found on the ventral abdomen. The remainder of the physical examination was unremarkable. Serum biochemistry results included mild hypernatremia (serum sodium concentration, 166 mmol/L; reference interval [RI], 151‐158 mmol/L), hyperglycemia (blood glucose concentration, 193 mg/dL; RI, 63‐118 mg/dL), increased serum albumin concentration (5.7 g/dL; RI, 2.2‐4.6 g/dL), and increased creatine kinase activity (17 136 IU/L; RI, 73‐260 IU/L). A CBC showed a normocytic, normochromic, non‐regenerative anemia (hematocrit, 20.3%; RI, 30%‐50%), thrombocytopenia (platelet count, 33 000/μL; RI, 180 000‐500 000/μL) with macroplatelets (mean platelet volume, 26.6 fl; RI, 9‐18 fl) and lymphocytosis (7440/μL; RI, 1000‐7000/μL). Blood smear analysis showed an estimated platelet count of approximately 30 000‐50 000 cells/μL with small numbers of platelet clumps.

### 2.4 Thoracic radiographs and abdominal ultrasonography

**FIGURE 1:**
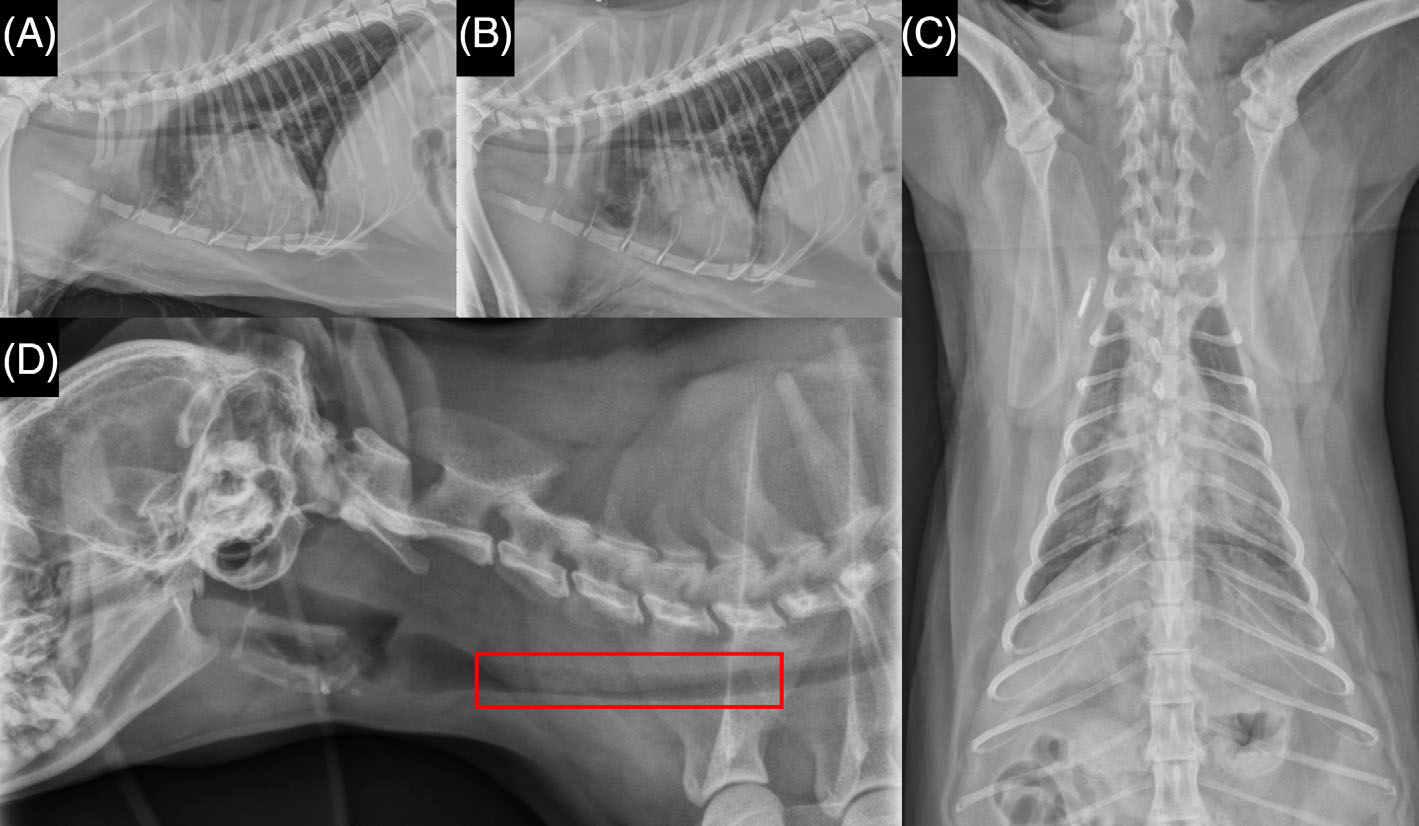
Day 1 thoracic radiograph imaging of cat patient. Left lateral (A) and right lateral (B) and dorsoventral (C) thoracic radiographs. Note moderate, patchy, alveolar pulmonary patterns throughout the ventral aspects of the right cranial and right middle lung lobes and cranial and caudal subsegments of the left cranial lung lobe; and mild widening of the cranial mediastinum by a soft tissue opacity without a discrete mass lesion. Right lateral cervical (D) radiographic image shows a visible dorsal tracheal membrane superimposed over most of the cervical tracheal luminal diameter (red box). The cardiac silhouette, pulmonary vasculature, nasopharynx, larynx, hyoid apparatus, musculoskeletal structures, and cranial portion of the abdomen were unremarkable.

Thoracic radiographs disclosed moderate, patchy, alveolar pulmonary patterns throughout the ventral aspects of the right cranial and right middle lung lobes and cranial and caudal subsegments of the left cranial lung lobe. Mild widening of the cranial mediastinum by a soft tissue opacity without a discrete mass lesion was observed (Figure 1). A visible dorsal tracheal membrane superimposed over most of the cervical tracheal luminal diameter was noted. The cardiac silhouette, pulmonary vasculature, nasopharynx, larynx, hyoid apparatus, musculoskeletal structures, and cranial portion of the abdomen were unremarkable on radiography. Given the suspected thrombopathy, the primary consideration was pulmonary hemorrhage into the dorsal tracheal membrane. No remarkable changes from Day 1 radiographs were observed on Day 2. Abdominal ultrasonography showed mesenteric lymphadenomegaly, likely of an inflammatory or neoplastic process, mild splenomegaly, extramedullary hematopoiesis, urinary bladder debris consistent with hematuria, gallbladder debris as a response to either cholestasis, colecystisis, or hemorrhage, and loculated fluid in the left inguinal region indicative of hemorrhage or seroma.

### 2.5 Patient management

Given suspicion of ongoing pulmonary and tracheal hemorrhage, the cat was hospitalized in the intensive care unit for further monitoring and treatment with 2 μg/kg desmopressin SC. Because fresh whole blood was unavailable at the time, the cat was transfused with 1 unit of type A and cross‐matched packed red blood cells. After transfusion, the cat was started on an IV infusion of epsilon‐aminocaproic acid (50 mg/kg bolus for the first hour then 15 mg/kg/hour for 12 hours). The cat was initially deemed stable and respiratory status had improved such that radiographic imaging was performed. On Day 2 of hospitalization, the cat developed worsening of stridor and stertor along with radiographic evidence of bilateral pulmonary infiltrates, suspected to be caused by progression of tracheal and pulmonary hemorrhage. The cat was provided supplemental oxygen (21%‐60%). A second SC 2 μg/kg dose of desmopressin was administered, and the cat was restarted on aminocaproic acid (50 mg/kg IV over an hour, q8h). On Day 3, the cat's respiratory signs resolved with only mild stridor noted. The cat was discharged for home care and monitoring along with tranexamic acid (162.5 mg PO q12h for 2‐3 days) for any future episodes and gabapentin (100 mg PO q8‐12h) as required to minimize anxiety. Given the signalment and spontaneous bleeding diatheses, a congenital platelet disorder of genetic etiology was suspected. Additional platelet function assays with subsequent genetic investigation and functional molecular experiment efforts were pursued.

### 2.6 Control samples

Whole blood EDTA venous blood samples from an additional cohort (n = 20) of apparently healthy geriatric (>10 years of age) client‐owned cats without prior history of bleeding diatheses and cleared of any cardiovascular disease were collected for genotyping. A clinically healthy 35‐month‐old male cat was included as a control; blood from this cat was collected for platelet aggregometry and flow cytometry analyses. An additional 3.2% sodium citrate venous blood sample from an age‐and sex‐matched cat was obtained for platelet isolation, RNA extraction, and subsequent end‐point RT‐PCR reactions.

### 2.7 Flow cytometry

To detect surface expression of αIIbβ3 integrin, platelet‐rich plasma (PRP) was generated from citrated whole blood by centrifugation at 200 × *g* for 5 minutes (acceleration 1, no break, room temperature). Platelet‐rich plasma was standardized to 1 × 10^7^ cells/mL with Tyrode‐4‐(2‐hydroxyethyl)piperazine‐1‐ethanesulfonic acid (Tyrode‐HEPES) buffer (pH 7.2, dextrose 5 mM, no divalent cations), followed by treatment with 20 μM adenosine diphosphate (ADP; Sigma‐Aldrich, St. Louis, MO, USA) or 0.005 U/mL thrombin (Haematologic Technologies, Essex Junction, VT, USA) for 15 minutes at 37°C. Platelet activation was measured by surface expression of platelet P‐selectin as previously described.^9^ The P‐selectin and αIIbβ3 proteins were labeled with fluorescein isothiocyanate‐conjugated rat anti‐mouse monoclonal antibodies to CD62P (1:200, clone: RB40.34; BD Pharmingen, San Jose, California, USA) and with allophycocyanin‐conjugated mouse anti‐human monoclonal antibodies to CD61 (1:1000, clone: VI‐PL2; eBioscience, San Diego, California, USA), respectively. After labeling for 45 minutes at 37°C, PRP was fixed in 1% paraformaldehyde for 30 minutes at room temperature and stored at 4°C for further analysis using flow cytometry.

Platelets were identified based on forward‐ and side‐scatter properties on log scale and CD61‐positive events. Gating for platelets was established by 0.3, 0.9, and 5 μm calibration beads and fluorescence‐minus‐1 controls. P‐selectin expression in the presence or absence of agonists was measured as percentage of CD62P‐positive events out of 10 000 events or median fluorescence intensity (MFI). Fluorescence spectral overlap was compensated by using isotype antibodies conjugated to fluorochromes on positive and negative calibration beads (BD Biosciences, Franklin Lakes, New Jersey, USA) and analyzed by flow cytometry under the same experimental conditions. All flow cytometry results were analyzed and compensation matrixes calculated using commercially available software (Flowjo v10.8; BD Biosciences, Franklin Lakes, New Jersey, USA).

### 2.8 Whole blood impedance platelet aggregometry

Platelet aggregometry was performed in duplicate using an automated whole blood impedance platelet aggregometer (Multiplate, Roche, Mannheim, Germany) according to the manufacturer's instructions. In brief, 300 μL of heparinized whole blood was first kept at room temperature for 30 minutes, followed by dilution in an equal volume of 0.9% sodium chloride at 37°C for 3 minutes. Diluted blood was subjected to constant shear rate created by an 800 rpm‐spinning Teflon‐coated magnetic stir bar. Adenosine diphosphate (6.25 μM; Millipore Sigma, Burlington, Massachusetts, USA) then was added, and electrical impedance recorded for 6 minutes. Platelet aggregation, measured as electrical impedance over time on a pair of silver‐coated electrodes, was reported as area under the curve (AUC), velocity (AU/min), and maximum aggregation (AU).

### 2.9 Whole blood gDNA isolation

Patient and validation cohort genomic DNA was isolated using Gentra Puregene Blood Kit according to the manufacturer's instructions (QIAGEN, Hilden, Germany). Quality and concentrations of DNA were quantified by spectrophotometry (NanoDrop One/One, Thermofisher, Waltham, GA, USA) and stored at −20°C for further use.

### 2.10 *ITGA2B* genotyping

**FIGURE 2:**
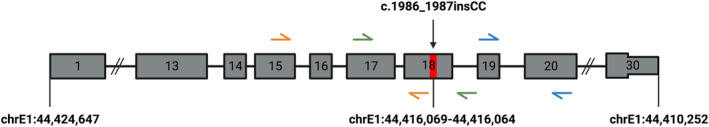
*ITGA2B* variant position and PCR primers. Illustration of *ITGA2B* gene body depicting constructed Sanger sequencing (green arrows) and RT‐PCR primers (orange [upstream] and blue [downstream]) are presented. Red mark on exon 18 depicts position of identified variant. chr, chromosome; ins, insertion; *ITGA2B*, *integrin alpha‐IIb*.

Given thrombocytopathia of presumed genetic etiology, the cat was genotyped for a previously identified *ITGA2B* (c.1986delC) variant in another cat with GT.^9^ A new primer set, flanking the entirety of ENSFCAG00000003056.6 exon 18, was designed using Primer3Plus software and specificity confirmed using University of California‐Santa Cruz's (UCSC) In‐Silico PCR tool on the felCat9 cat genome build. Specific confirmed DNA oligonucleotides (F 5′‐GCGGGTGCTACTGCTGAAT‐3′, R 5′‐TGAAAAGGAGTTTGGAGCTGA‐3′) were synthesized by Integrated DNA Technologies (IDT; CoralVille, Iowa, USA; Table 1; Figure 2). Amplification of PCR targets was conducted via end‐point PCR using the TaKaRa LA Taq with GC Buffer PCR Kit. A single 25 μL PCR reaction consisted of 0.25 μL of TaKaRa LA Taq, 12.5 μL of 2X GC Buffer II, 4 μL of dNTP (2.5 mM), 0.5 μL of both forward and reverse primers (20 μM), 1‐2 μL of DNA template (>20 ng/μL), and 5.25‐6.25 μL of laboratory grade sterilized distilled water. The PCR reactions were carried out using a Mastercycler Nexus (Eppendorf, Hamburg, Germany) under the following conditions: 94°C for 1 minute, 30 cycles of 94°C denaturation for 30 seconds, 56°C annealing for 30 seconds, 72°C extension for 2 minutes, and a final extension at 72°C for 5 minutes. Ten microliters (+2 μL of loading dye) of the resulting PCR products were loaded on a 1% agarose gel containing 1‐2 μL of SYBR Safe (Thermofisher, Waltham, GA, USA) and run on a PowerPac Basic (Bio‐Rad, Hercules, California, USA) at a constant 120 V for 30‐45 minutes. Primer set specificity and expected amplicon sizes were confirmed by EPI‐blue gel imaging with an automated imaging system (Azure300, Azure Biosystems, Dublin, California, USA). A PCR clean‐up step was performed for all samples with the ExoSAP‐IT Express Kit (Thermofisher, Waltham, GA, USA) according to the manufacturer's protocol. Treated PCR products and their corresponding primer set later were diluted according to facility specifications and submitted for Sanger sequencing (UC Davis, DNA Sequencing Facility, Davis, California, USA). Chromatograms of the resulting .ab1 files were manually inspected and analyzed with using Sequencher (Gene Codes, Ann Arbor, Michigan, USA) and SnapGene Viewer sequence analysis software (https://www.snapgene.com/).

**TABLE 1:** Sanger sequencing genotyping & RT‐PCR primer specifications.

Primer name	Primer sequence	GC%	Exon Target(s)	Tm (°C)	Product size
PCR					
*ITGA2B*_Genotyping_F	GCGGGTGCTACTGCTGAAT	57.9	18	56	676
*ITGA2B*_Genotyping_R	TGAAAAGGAGTTTGGAGCTGA	42.9			
RT‐PCR					
*ACTB*_F	ATGAGGCCCAGAGCAAGAG	57.9	2–3	61	510
*ACTB*_R	CTTCTCCAGGGAGGACGAG	63.2			
*ITGA2B*_Upstream_F	CACTGAATCCTGCTGTGAAGA	47.6	15‐18	61	255
*ITGA2B*_Upstream_R	CTCAGCTTGTCCCGGAAGT	57.9			
*ITGA2B*_Downstream_F	GCGGAGAGGATGATCTGTGT	55	19‐20	63	129
*ITGA2B*_Downstream_R	CCTCATGTAGTGGGCACCTG	60			

Abbreviations: *ACTB*, *beta (β)‐actin*; GC%, guanine‐cytosine percentage; *ITGA2B*, *integrin alpha‐IIb*; Tm, melting temperature.

### 2.11 Platelet RNA isolation

Citrated whole blood from the patient and a sex‐ and age‐matched clinically healthy control cat was incubated at 37°C for 30 minutes before PRP was generated by centrifugation as described above. The resulting PRP from the 2 samples from each subject were later amalgamated; a 100 μL aliquot from each sample was used to confirm the absence of substantial leukocyte contamination using an automated hematology analyzer (HM5, Zoetis, Parsippany‐Troy Hills, New Jersey, USA). Washed platelets from PRP were generated via a second centrifugation step (15 minutes, at 271 × *g*) and the platelet pellet was subjected to platelet RNA extraction using the AllPrep DNA/RNA/Protein Mini Kit (QIAGEN, Hilden, Germany) according to the manufacturer's protocol. Platelet RNA quality and concentrations were quantified by spectrophotometry (NanoDrop One/One, Thermofisher, Waltham, GA, USA) and stored at −80°C until further use.

### 2.12 cDNA synthesis and end‐point RT‐PCR

Sixteen microliters of patient and control RNA was used for DNA digestion and cDNA synthesis; double volume reactions were carried out according to the manufacturer's instructions using the SuperScript IV VILO Master Mix with ezDNase Enzyme Kit (Thermofisher, Waltham, GA, USA).

Variant effect on platelet *ITGA2B* transcript was verified via end‐point RT‐PCR. Primer sets for *beta (β)‐actin* (*ACTB*; housekeeper) and up‐ and downstream of the newly identified *ITGA2B* variant were constructed using the aforementioned methods (Table 1; Figure 2). A single 25 μL PCR reaction consisted of 0.25 μL of TaKaRa LA Taq, 12.5 μL of 2X GC Buffer II, 4 μL of dNTP (2.5 mM), 0.5 μL of both forward and reverse primers (20 μM), 2 μL (1.48 ng) of the resulting cDNA template, and 5.25 μL of laboratory grade sterilized distilled water. The RT‐PCR reactions were carried out by a Mastercycler Nexus (Eppendorf, Hamburg, Germany) under the following conditions: 94°C for 1 minute, 30 cycles of 94°C denaturation for 30 seconds, 61‐63°C annealing for 30 seconds, 72°C extension for 2 minutes, and a final extension at 72°C for 5 minutes. Ten μL (+2 μL of loading dye) of the resulting RT‐PCR products were loaded on a 1% agarose gel containing 1 μL of SYBR Safe (Thermofisher, Waltham, GA, USA) and run on a PowerPac Basic (Bio‐Rad, Hercules, California, USA) at a constant 80 V for 40‐50 minutes. EPI‐blue gel imaging for the resulting gel was performed using an Azure300 imaging system (Azure Biosystems, Dublin, California, USA). Gel images were analyzed using GelAnalyzer Software (v19.1).

## 3 RESULTS

### 3.1 Whole blood impedance platelet aggregometry

**FIGURE 3:**
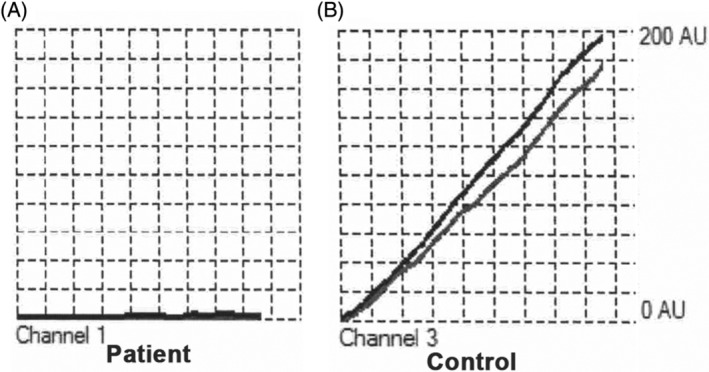
Whole blood impedance platelet aggregometry. Diluted whole blood was activated in the presence of 6.25 μM ADP; electrical impedance because of platelet aggregation was measured over 6 minutes in duplicate (black and gray lines on each graph displayed). Aggregation response was absent in the affected cat (A) compared to a control cat (B). AU, aggregation units; ADP, adenosine diphosphate.

Whole blood impedance platelet aggregometry showed that platelets from the patient did not aggregate in response to ADP (AUC = 0, velocity = 0 AU/min, maximum aggregation = 0 AU) compared to results generated in a healthy control cat as shown in Figure 3.

### 3.2 Flow cytometry

**FIGURE 4:**
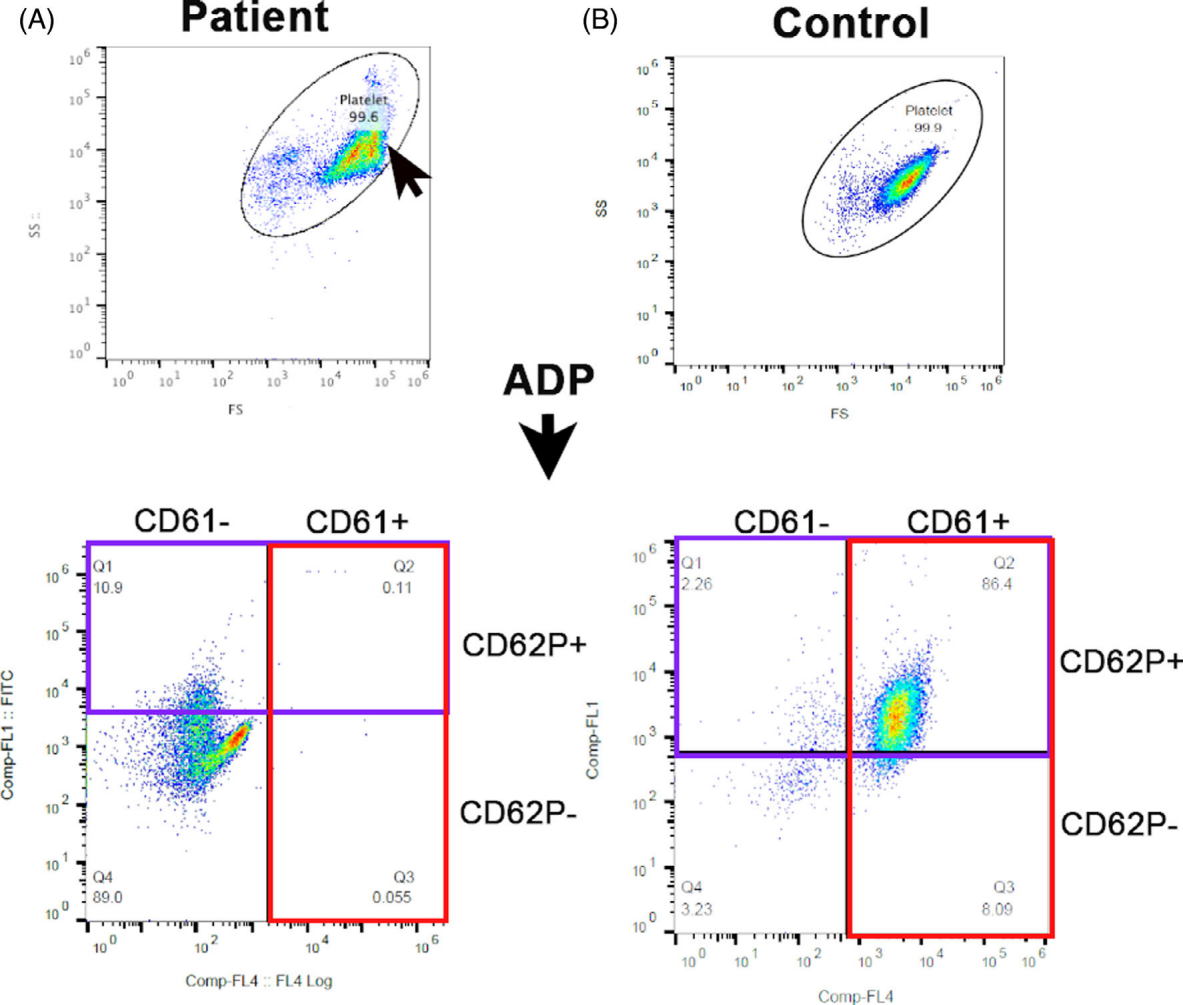
Representative scatter dot plots of platelet flow cytometry in the patient and a control cat. Platelet gate (oval) was established using 3 and 5 μm calibration beads. (A) Note the shift in forward‐scatter (FS) in the patient (arrow) indicating an increase in platelet size compared to the control cat. Platelets were activated with ADP and labeled with fluorophore‐conjugated antibodies to αIIbβ3 (CD61; red box) and P‐selectin (CD62P, purple box). (A) A complete absence of CD61 expression (red box) was noted in the patient compared to the control cat. (B) Most platelets (86.4%) in the control cat had externalized P‐selectin (CD62+) following exposure to ADP. (A) Only a portion (10.9%) of platelets in the affected cat had externalized P‐selectin upon ADP activation. ADP, adenosine diphosphate; FS, forward‐scatter; SS, short‐scatter.

Representative scatter dot plots of PRP generated in the patient and a control cat on flow cytometry are shown in Figure 4. Compared to the control cat, a shift in forward scatter property was noted indicating an increase in platelet size. No platelets expressed integrin subunit beta‐3 (CD61) of the surface integrin αIIbβ3 in the affected cat compared with the control cat, which showed that 95% of platelets expressed CD61 of the αIIbβ3 receptor (0% vs 95%). Platelets from the affected cat also had decreased alpha‐granule secretion in response to the agonist (ADP) as shown by decreased expression of surface P‐selectin (CD62P; 10.9% vs 86.4%). Results of flow cytometry further confirmed failure of platelets to aggregate in response to ADP because of complete absence of αIIbβ3 on the membrane surface.

### 3.3 *ITGA2B* genotyping

**FIGURE 5:**
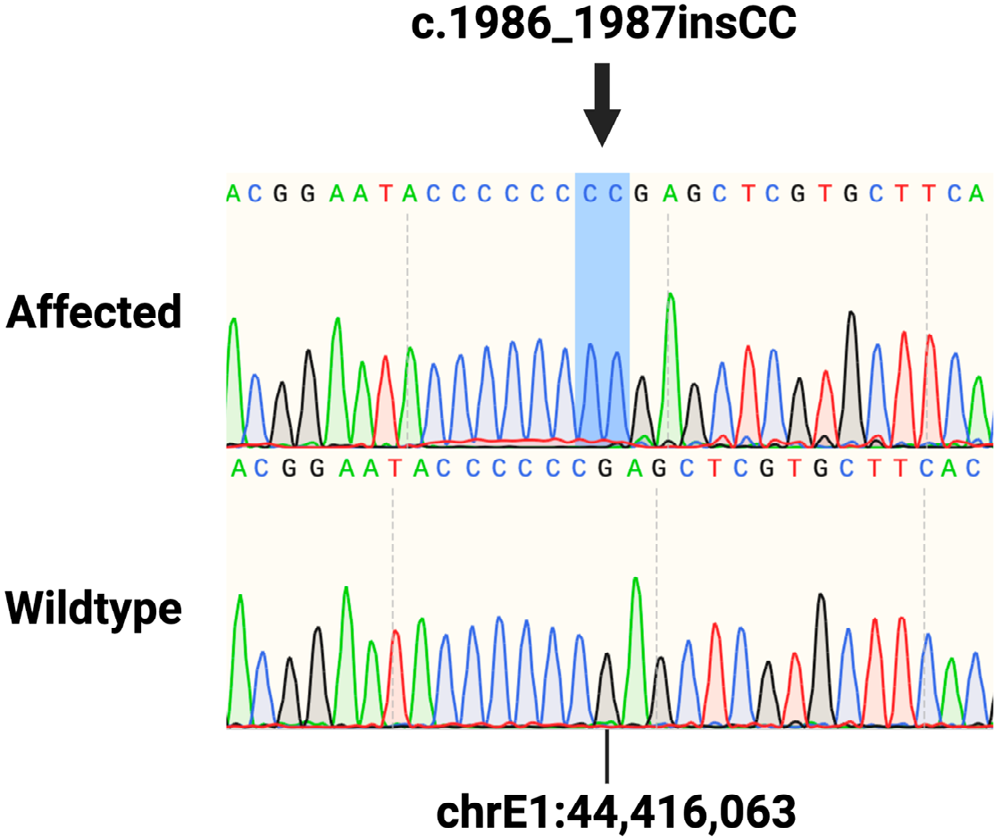
Sanger sequencing of the *ITGA2B* (c.1986_1987insCC) frameshift variant. Sanger sequencing chromatograms of the affected cat (top) homozygous for the double cytosine insertion (highlighted in blue) and a clinically healthy geriatric unrelated cat (bottom) are presented. chr, chromosome; ins, insertion; *ITGA2B*, *integrin alpha‐IIb*.

Patient genotyping for the previously reported c.1986delC variant for feline GT (Figure 5) identified a novel double cytosine insertion in exon 18 of the *ITGA2B* gene. This new c.1986_1987insCC and the previously reported variant are positioned within chrE1:44 416 064‐44 416 069 of the felCat9 gene annotation and each cause a frameshift with an implicated premature stop codon in the gene's 699th amino acid position. Sanger sequencing genotyping for the c.1986_1987insCC variant in a validation cohort comprised of 20 clinically healthy geriatric cats was performed (sequencing files available: https://doi.org/10.25338/B8JW72). Neither the variant, nor any other relevant variants, were found within the amplicon for any of these additionally genotyped cats. The variant was further screened in an additional 194 unphenotyped whole genome‐sequenced cats spanning 44 different breeds (99 Lives Sequencing Consortium; http://felinegenetics.missouri.edu/99lives). Screening did not identify cats harboring the *ITGA2B* c.1986_1987insCC variant.

### 3.4 End‐point RT‐PCR

**FIGURE 6:**
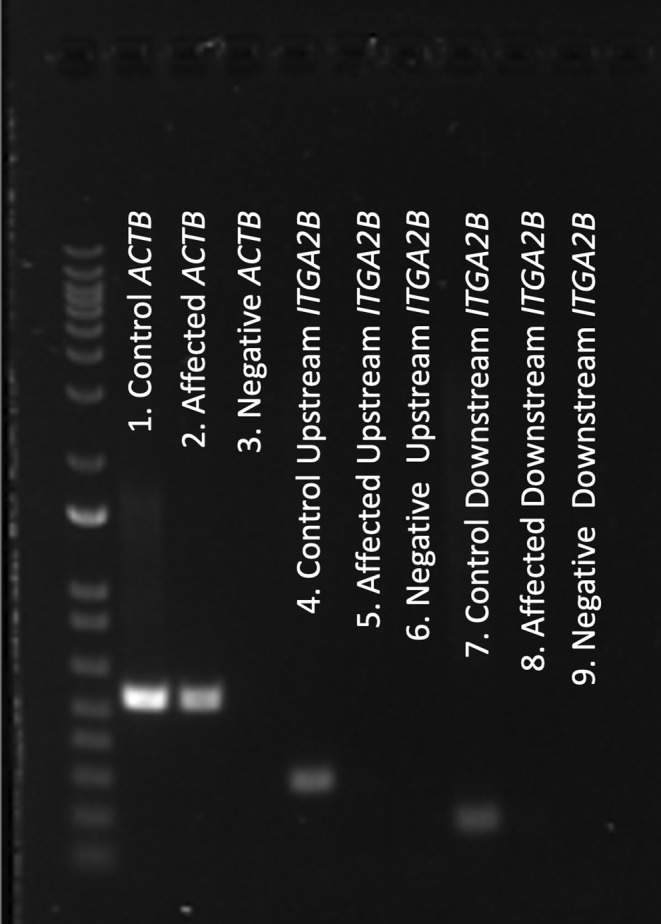
RT‐PCR reveals c.1986_1987insCC leads to complete null *ITGA2B* expression. Gel image of ladder (left) and the resulting RT‐PCR products are presented. *ACTB* housekeeper amplification was confirmed in a sex‐ and age‐matched clinically healthy cat (lane 1) and affected patient (lane 2) (bands between 650 and 500 bp marker). *ITGA2B* expression up‐ and downstream of the variant position (bands between 300‐200 bp and 200‐100 bp marker, respectively) was detected in the control cat (lane 4 and lane 7, respectively), but not in the affected cat (lane 5 and lane 8, respectively). Negative‐control samples for the *ACTB* and 5′ and 3′ *ITGA2B* RT‐PCR reactions are displayed (lane 3, lane 6, and lane 9, respectively). Original gel image is provided in Figure S1. *ACTB*, *beta (β)‐actin*; bp, base pair; *ITGA2B*, *integrin alpha‐IIb*.

Definitive variant effect on *ITGA2B* platelet transcript expression was verified by RT‐PCR. Successful RNA extraction and cDNA synthesis was confirmed with *ACTB* transcript bands for both the patient and the sex‐ and aged‐matched clinically healthy cat. *ITGA2B* expression on platelets also was validated in the control sample. *ITGA2B* expression up‐ and downstream of the variant was present in the control sample but absent in the affected cat, accounting for complete null protein expression as observed in the patient's flow cytometry data. Amplicon bands were congruent with expected in silico PCR products. Secondary gel bands suggestive of non‐specific binding were not observed (Figure 6).

## 4 DISCUSSION

Similarities observed between humans and dogs with thrombopathia prompted a candidate gene approach for investigating genetic variants in *ITGA2B* in a cat with severe bleeding diathesis. Initial screening by Sanger genotyping for the c.1986delC variant previously was performed by the authors in a cat with GT.^9^ This variant however was not observed in the affected cat of the present report and instead we identified a new variant in the same genomic position. This new c.1986_1987insCC variant is a double cytosine insertion positioned within chrE1:44 416 064‐44 416 069 of the felCat9 genome reference. This variant was not observed in any of the 20 unrelated clinically healthy geriatric cats in our validation cohort nor in any of the unrelated cats within the 99 Lives Sequencing Consortium (http://felinegenetics.missouri.edu/99lives). Although we were not able to obtain blood samples from the tom and queen of the affected patient, interpretation of sequencing chromatograms suggests that the patient has 2 copies of the null *ITGA2B* allele, which supports an autosomal recessive MOI consistent with all other reported cases of GT in mammals. The insertion leads to a frameshift near the 3′ end of the *ITGA2B* transcript. Null 5′ and 3′ *ITGA2B* transcript expression within the patient's platelets was confirmed by RT‐PCR, supportive of nonsense‐mediated decay (NMD), a eukaryotic compensatory surveillance pathway for RNA quality control and gene regulation, as an explanation for complete absence of alpha‐IIb (CD41a), and thus αIIbβ3 expression. It is speculated that the chrE1:44 416 064‐44 416 069 locus in cats is particularly susceptible to de novo mutations. Although not a repetitive region, the chrE1:44 416 064‐44 416 069 sequence of ENSFCAG00000003056.6 is composed of 6 tandem cytosines, making this locus a target for polymerase slippage, and therefore prone to spontaneous removal or addition of cytosines that would lead to pathogenic effects on *ITGA2B* expression and protein function. Glanzmann's thrombasthenia is rare in humans and dogs with previous studies documenting rare frequency of causal variants in each species along with evidence of conserved sequences between the functional domains of canine and human *ITGA2B*.^9^, ^11^, ^12^, ^13^, ^14^ The prevalence of GT in cats is believed to be very low because the c.1986_1987insCC *ITGA2B* variant reported here was not found in any of the cats in the large screening cohort. Additional large‐scale screening efforts however are necessary to assess the prevalence of this variant in the general cat population.

Alpha‐IIb (CD41) and beta‐3 (CD61) subunits constitute the αIIbβ3 integrins, the most abundant transmembrane glycoprotein expressed on platelet surfaces.^15^, ^16^, ^17^ In response to platelet agonists, the activation of αIIbβ3 is initiated by cytoplasmic signaling cascades, which in turn lead to a conformational change of its transmembrane domains from a bent to straight configuration, a process called inside‐out signaling.^15^, ^18^ This signaling process leads to an increased affinity of the receptor's integrin head domains for ligand binding by fibrinogen or other soluble adhesive proteins. This process is essential for the regulation of platelet adhesion and platelet‐to‐platelet interactions, extracellular matrix assembly, and mechanotransduction in thrombosis.^19^, ^20^ The pathogenic effect of the identified null *ITGA2B* variant explains the absence of the beta‐3 subunit (CD61) on flow cytometry because failure of integrin assembly in the endoplasmic reticulum inhibits further export to the cell surface by the Golgi apparatus. The beta‐3 subunit also represents a known component of the vitronectin receptor on platelet surfaces in some species.^21^, ^22^, ^23^ To our knowledge, the vitronectin receptor has not been specifically investigated in cats. Thus, failure to detect beta‐3 by flow cytometry in this cat suggests a species difference or reflects failure of our selected antibody to cross‐react with feline vitronectin‐associated beta‐3.

Fundamental differences are present between our cat's phenotype and the previously reported cat with GT. First, this variant alone does not explain the observed macrothrombocytopenia in our patient. Although the effects of null or hypomorphic αIIbβ3 have been studied in hemostasis, its relationship to platelet numbers and morphology previously have been questioned in mouse and dog models.^24^, ^25^, ^26^ Previous studies have reported compound heterozygosity in human patients with both the GT and macrothrombocytopenia phenotypes.^27^, ^28^ However, given that our patient was homozygous for the c.1986_1987insCC variant, compound heterozygosity can be ruled out. Nevertheless, another unidentified variant in this cat may have independently contributed to the observed macrothrombocytopenia. In addition, other causes such as immune‐mediated destruction or pre‐mature platelet clearance also may explain our patient's thrombocytopenia with increased platelet size because of thrombopoiesis. Whether the c.1986_1987insCC variant in cats leads to a pleiotropic effect on decreased platelet number, aberrant morphology, as well as function remains a question that may be further elucidated using whole genome sequencing and continued clinical experience with cats harboring this variant. At minimum, the variant reported here explains the GT phenotype.

In summary, we report the results from a candidate gene approach for investigating the underlying genetic etiology of thrombocytopathia and macrothrombocytopenia in a cat. Our research efforts resulted in the identification of a novel autosomal recessive de novo variant in the *ITGA2B* gene that leads to complete loss of transcript expression. To our knowledge, this case is the second reported pathogenic variant for GT in cats. Additionally, our study provides a small‐volume, standardized, successful protocol for adequate platelet RNA isolation and subsequent molecular genetic assessment of gene expression in cats. Our results shed light on the association and enigmatic phenotype of macrothrombocytopenia with GT. Additional genetic studies are warranted to exclude other possible genetic variants that might explain this cat's complete thrombopathy phenotype.

## CONFLICT OF INTEREST DECLARATION

Joshua A. Stern serves as Associate Editor for the Journal of Veterinary Internal Medicine. He was not involved in review of this manuscript. No other authors declare a conflict of interest.

## OFF‐LABEL ANTIMICROBIAL DECLARATION

Authors declare no off‐label use of antimicrobials.

## INSTITUTIONAL ANIMAL CARE AND USE COMMITTEE (IACUC) OR OTHER APPROVAL DECLARATION

Approved by the University of California‐Davis (Davis, CA, USA) IACUC (protocol #20095 and #21857).

## HUMAN ETHICS APPROVAL DECLARATION

Authors declare human ethics approval was not needed for this study.

## Supporting information


**Figure S1:** RT‐PCR reveals c.1986_1987insCC leads to complete null *ITGA2B* expression. Intact image of ladder (left) and the resulting RT‐PCR products are presented. *ACTB* housekeeper amplification was confirmed in a sex‐ and age‐matched clinically healthy cat (lane 1) and affected patient (lane 2) (bands between 650 and 500 bp marker). *ITGA2B* expression up‐ and downstream of the variant position (bands between 300‐200 bp and 200‐100 bp marker, respectively) was detected in the control cat (lane 4 and lane 7, respectively), but not in the affected cat (lane 5 and lane 8, respectively). Negative‐control samples for the *ACTB* and 5′ and 3′ *ITGA2B* RT‐PCR reactions are displayed (lane 3, lane 6, and lane 9, respectively). *ACTB*, *beta (β)‐actin*; bp, base pair; *ITGA2B*, *integrin alpha‐IIb*.
