# Supplementary material for: A novel ITGA2B double cytosine frameshift variant (c.1986_1987insCC) leads to Glanzmann's thrombasthenia in a cat
Source: J Vet Intern Med. 2024 Mar 1;38(3):1408–17. doi: 10.1111/jvim.17030 (PMC11099703; doi:10.1111/jvim.17030)
Supplement: Supplementary file 1 — Figure S1: RT‐PCR reveals c.1986_1987insCC leads to complete null ITGA2B expression. Intact image of ladder (left) and the resulting RT‐PCR products are presented. ACTB housekeeper amplification was confirmed in a sex‐ and age‐matched clinically healthy cat (lane 1) and affected patient (lane 2) (bands between 650 and 500 bp marker). ITGA2B expression up‐ and downstream of the variant position (bands between 300‐200 bp and 200‐100 bp marker, respectively) was detected in the control cat (lane 4 and lane 7, respectively), but not in the affected cat (lane 5 and lane 8, respectively). Negative‐control samples for the ACTB and 5′ and 3′ ITGA2B RT‐PCR reactions are displayed (lane 3, lane 6, and lane 9, respectively). ACTB, beta (β)‐actin; bp, base pair; ITGA2B, integrin alpha‐IIb. [file JVIM-38-1408-s001.pdf]

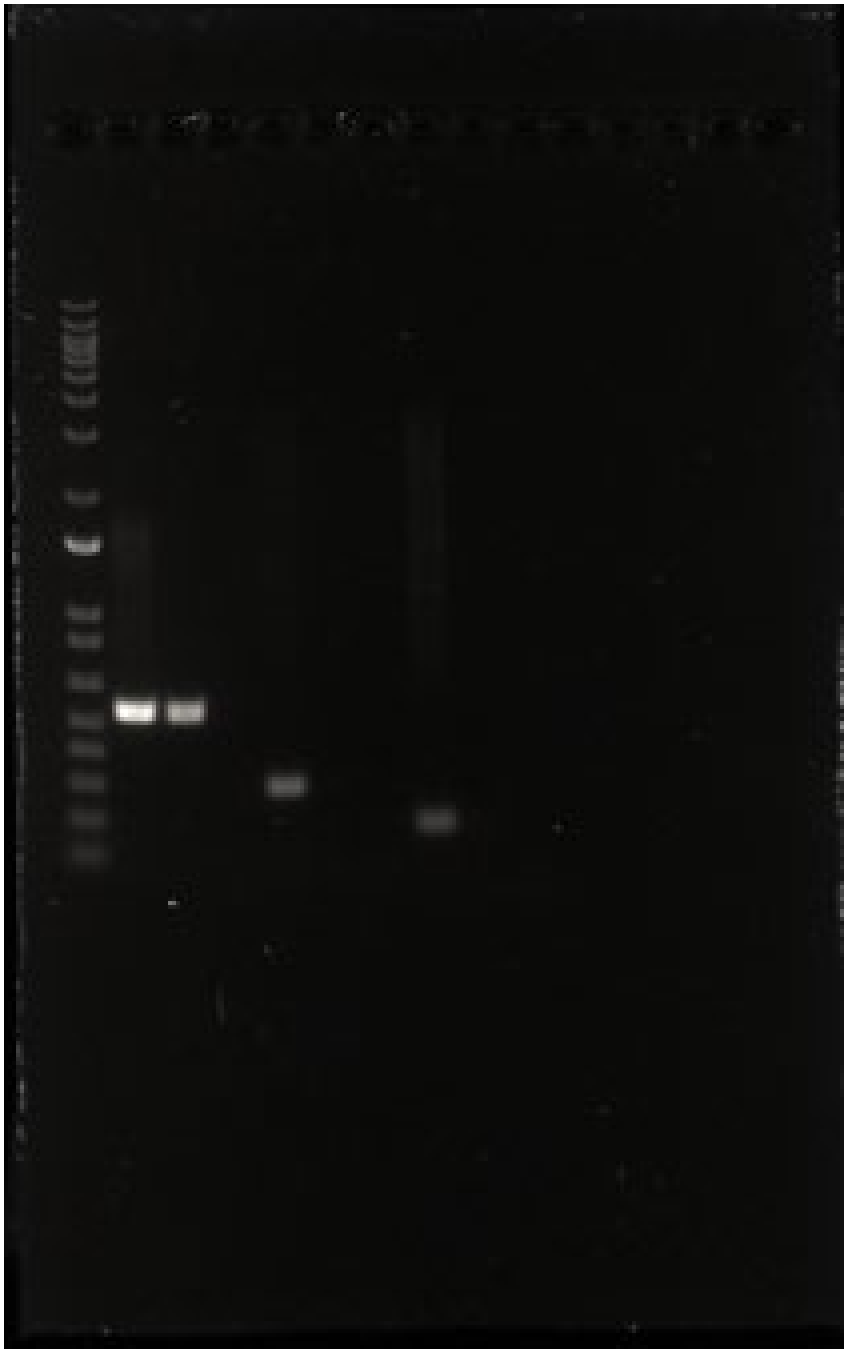

Supplemental Figure 1. RT-PCR reveals c.1986_1987insCC leads to complete null *ITGA2B* expression. Intact image of ladder (left) and the resulting RT-PCR products are presented. *ACTB* housekeeper amplification was confirmed in a sex- and age-matched clinically healthy cat (lane 1) and affected patient (lane 2) (bands between 650-500 bp marker). *ITGA2B* expression up- and downstream of the variant position (bands between 300-200 bp and 200-100 bp marker, respectively) was detected in the control cat (lane 4 and lane 7, respectively), but not in the affected cat (lane 5 and lane 8, respectively). Negative-control samples for the *ACTB* and 5’ and 3’ *ITGA2B* RT-PCR reactions are displayed (lane 3, lane 6, and lane 9, respectively).

Abbreviations: *ACTB = beta (β)-actin*, bp = base pair, *ITGA2B = integrin alpha-IIb*.
